# Supplementary material for: Enhanced control of self-doping in halide perovskites for improved thermoelectric performance
Source: Nat Commun. 2019 Dec 17;10:5750. doi: 10.1038/s41467-019-13773-3 (PMC6917797; doi:10.1038/s41467-019-13773-3)
Supplement: Supplementary file 1 — Supplementary Information [file 41467_2019_13773_MOESM1_ESM.pdf]

Supplementary Information for

Enhanced control of self-doping in halide perovskites for improved thermoelectric performance

Tianjun Liu,[1,2] Xiaoming Zhao,[2] Jianwei Li,[3] Zilu Liu[3], Fabiola Liscio,[4] Silvia Milita,[4] Bob C Schroeder[3] and Oliver Fenwick.[1,2]*

[1] School of Engineering and Material Sciences, Queen Mary University of London, Mile End Road, E1 4NS, United Kingdom.

[2] The Organic Thermoelectrics Laboratory, Materials Research Institute, Queen Mary University of London, Mile End Road, London, E1 4NS, United Kingdom.

[3] Department of Chemistry, University College London, 20 Gordon Street, London, WC1H 0AJ, United Kingdom.

[4] Istituto per la Microelettronica e Microsistemi (IMM)-Consiglio Nazionale delle Ricerche (CNR), Via Gobetti 101, 40129 Bologna, Italy.

Corresponding author: Oliver Fenwick (o.fenwick@qmul.ac.uk)

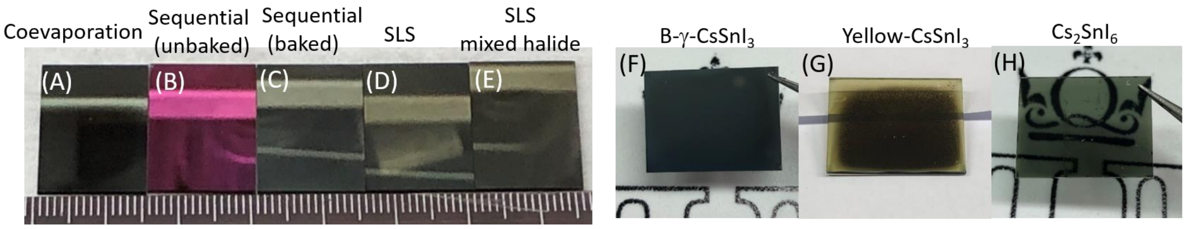

Supplementary Figure 1. Optical images of $CsSnI_3$ and mixed halide $CsSnI_{3-x}Cl_x$ thin films with different deposition methods (A) to (E). The degradation images of films exposed to air (F) to (H).

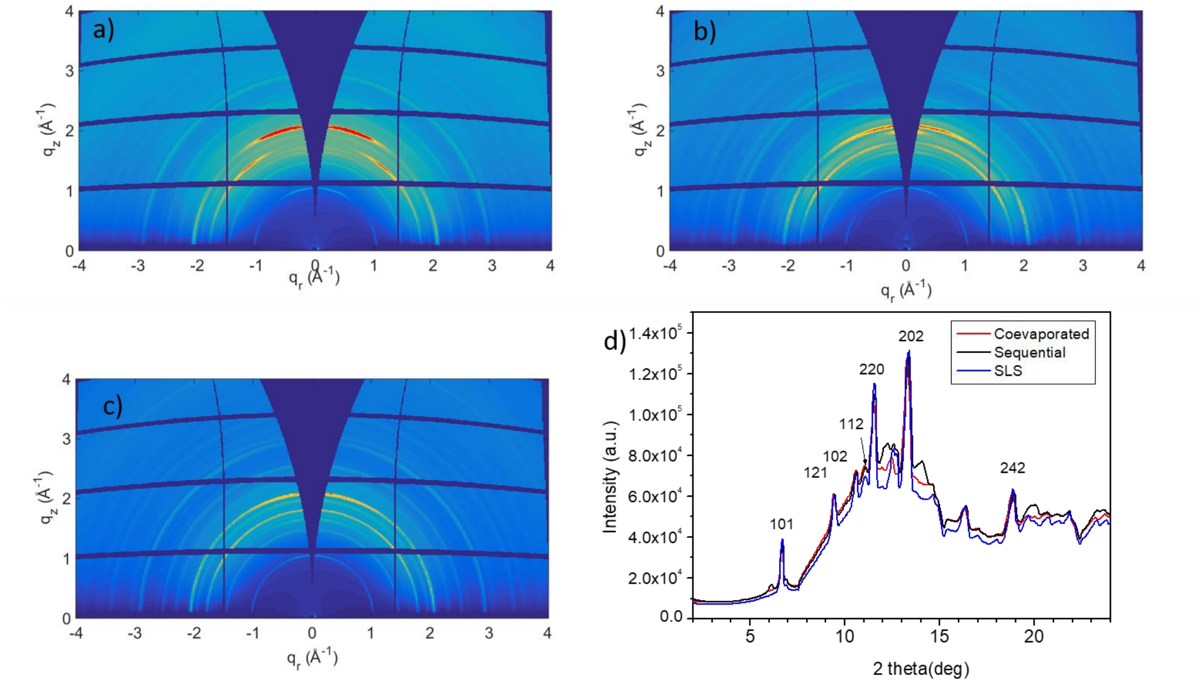

Supplementary Figure 2. 2D GIXRD images of a) coevaporated, b) sequential and c) SLS films recorded at an angle of incidence, $\alpha_i = 0.5°$. d) Diffracted intensities integrated along the scattering angles. The peaks are marked with the Miller indices of the B-γ-$CsSnI_3$ phase.

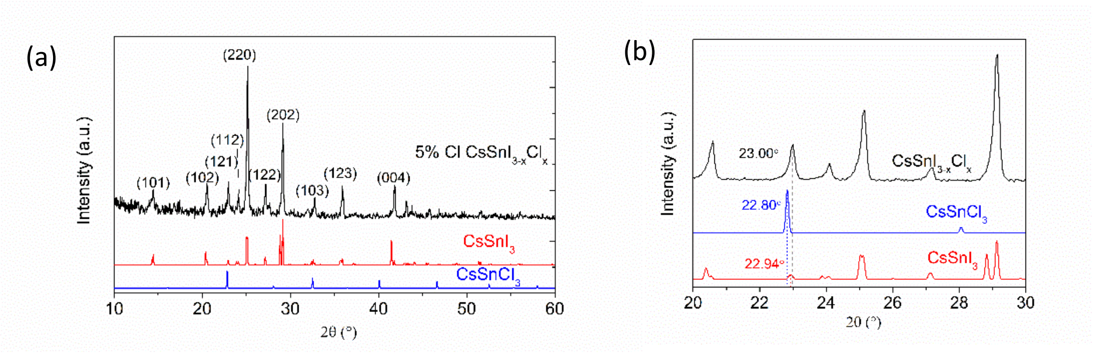

Supplementary Figure 3. XRD spectrum of an SLS 5% mixed halide $CsSnI_{3-x}Cl_x$ thin film. (a) Scanning angle from 10° to 60° with rate of 5°/minute. (b) Slow scan with rate of 1°/minute from 20° to 30°.

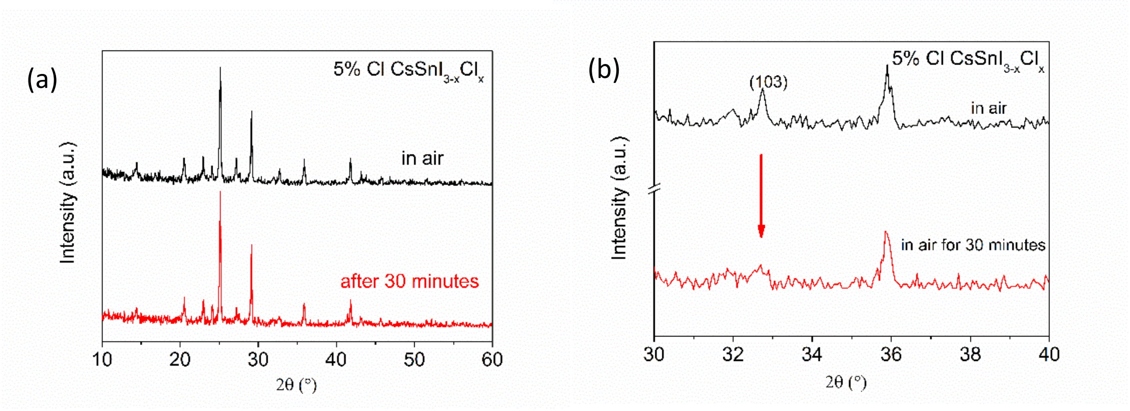

Supplementary Figure 4. XRD spectrum of SLS 5% mixed halide $CsSnI_{3-x}Cl_x$ thin films after air exposure for 30 minutes from 10° to 60° (a) and 30° to 40° (b).

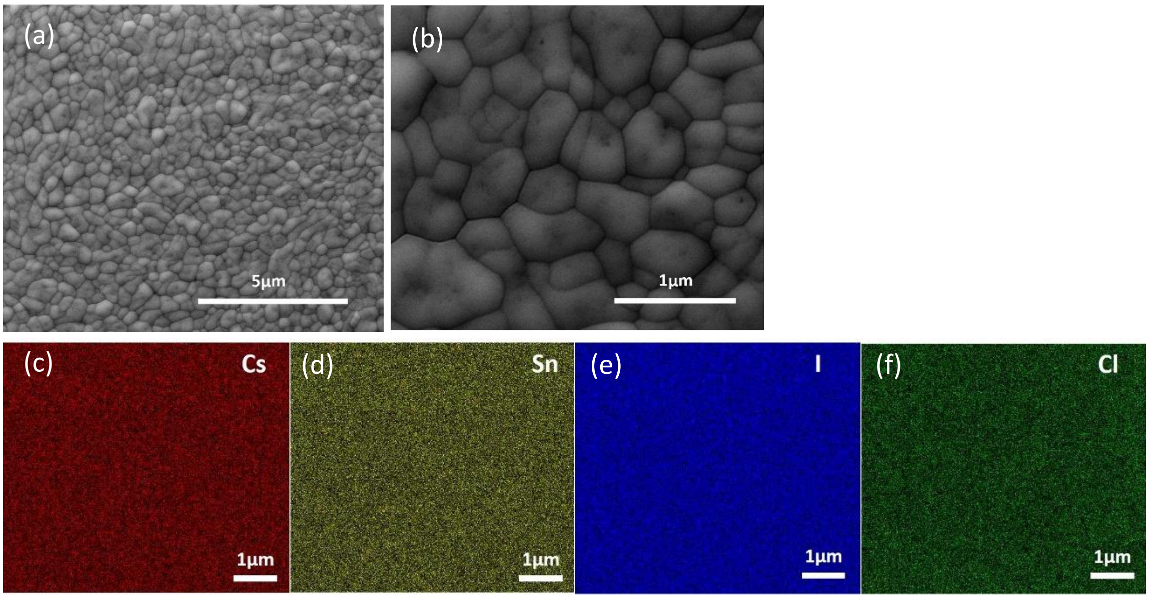

Supplementary Figure 5. (a), (b) SEM images of a 5% $SnCl_2$ mixed halide $CsSnI_{3-x}Cl_x$ film with thickness of 250 nm. (c), (d), (e) and (f) energy dispersive spectrum (EDS) based on (a) with element mapping of Cs, Sn, I and Cl, respectively. 5%-Cl mixed halide perovskite $CsSnI_{3-x}Cl_x$ films with 250nm thickness present sub-micrometre grain size in SEM images. Elemental mapping by energy dispersive X-ray spectroscopy (EDS) in (f) shows that Cl is uniformly distributed in the mixed halide perovskite films.

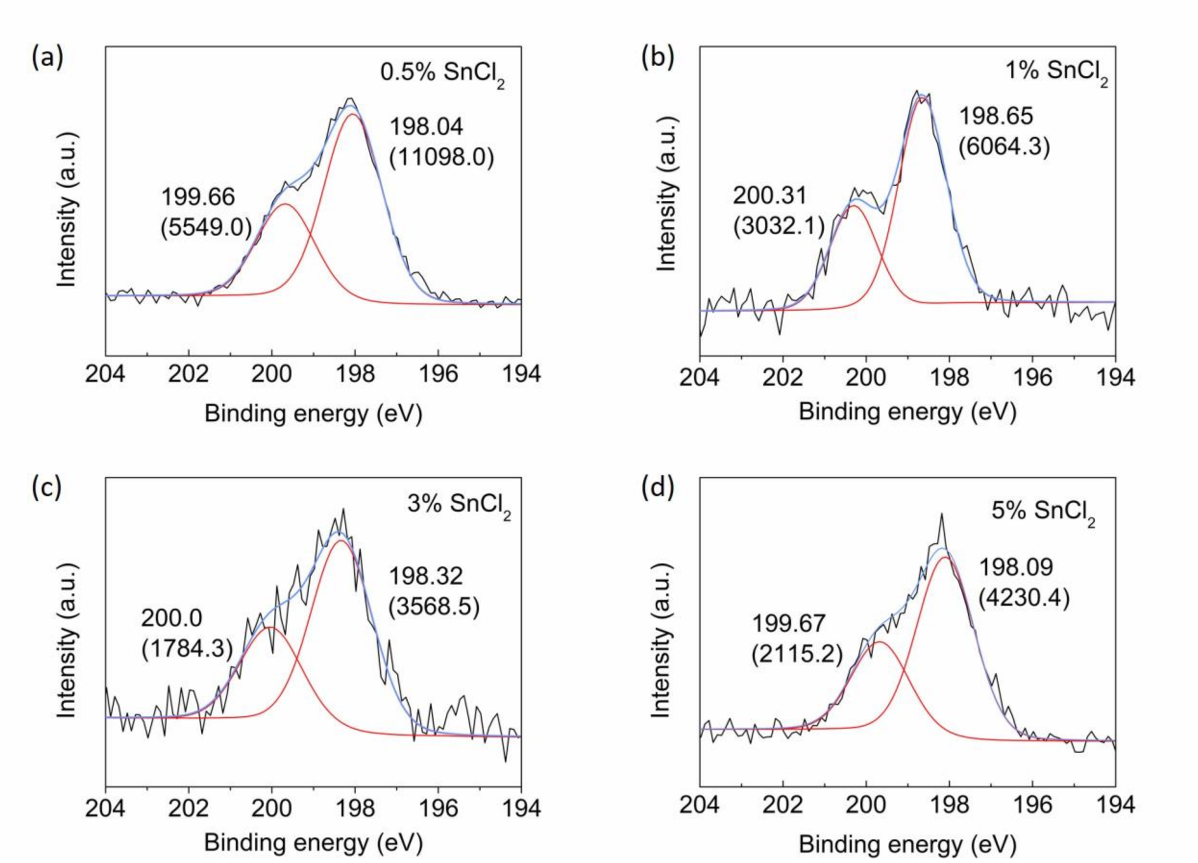

Supplementary Figure 6. XPS spectra of Cl 2*p* in mixed halide $CsSnI_{3-x}Cl_x$ samples of (a) 0.5%, (b) 1%, (c) 3% and (d) 5% $SnCl_2$.

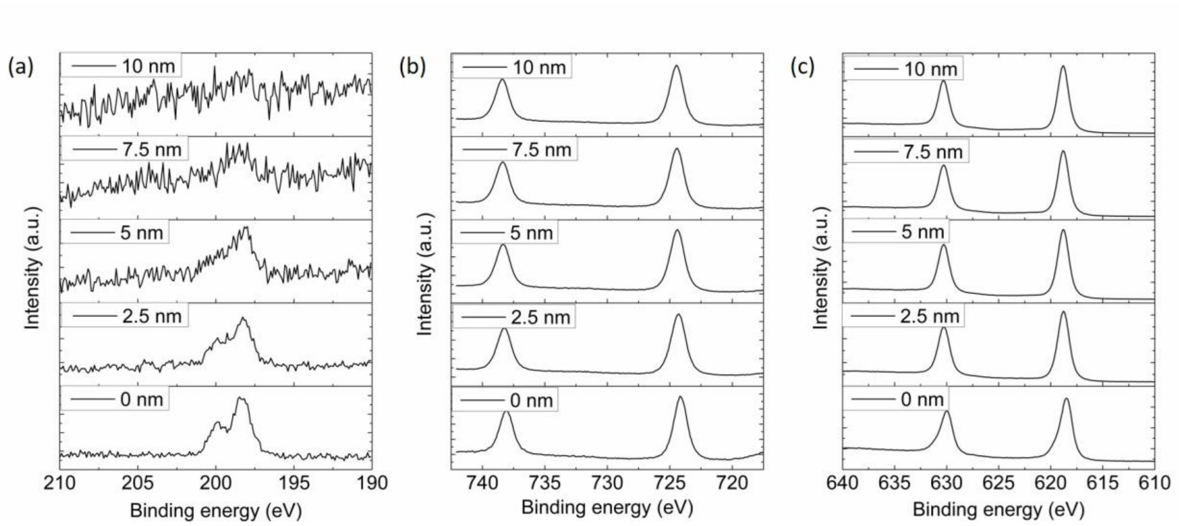

Supplementary Figure 7. XPS depth profile from 0 to 10 nm of Cl 2*p* (a), Cs 3*d* (b) and I 3*d* (c).

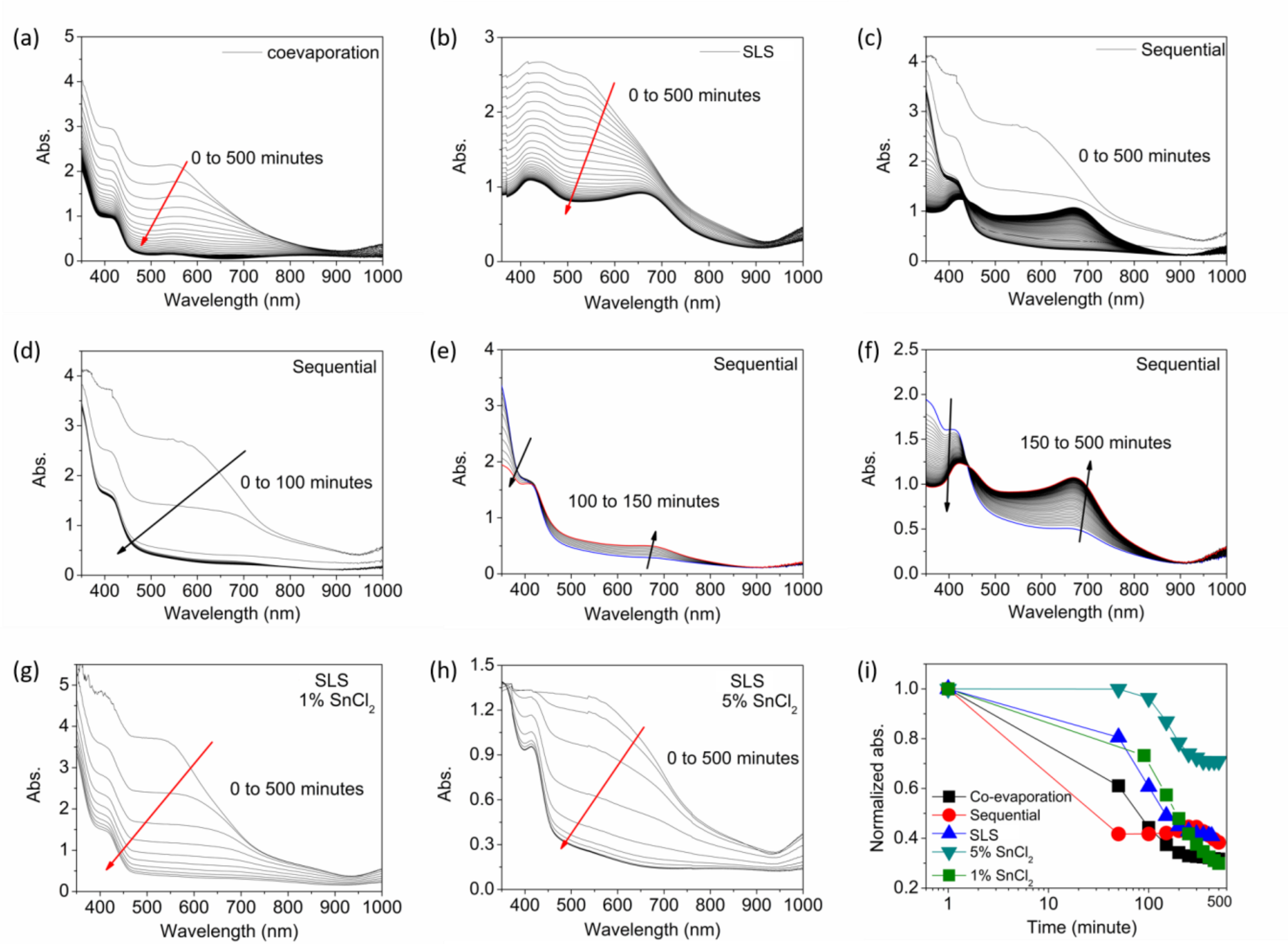

Supplementary Figure 8. UV-vis absorption spectra of $CsSnI_3$ films formed from three different deposition methods: co-evaporation (a), SLS (b) and sequential (c). The spectra of sequentially deposited films have been divided to three parts with time from 0 to 100 minutes (d), 100 to 150 minutes (e) and 150 to 500 minutes (f). UV-vis absorption spectra of 1% $SnCl_2$ (g) and 5% $SnCl_2$ (h) mixed halide $CsSnI_{3-x}Cl_x$ thin films by SLS method. The measurement in ambient air took 500 minutes. (i) Time dependent absorbance (normalized) at 420 nm of different films.

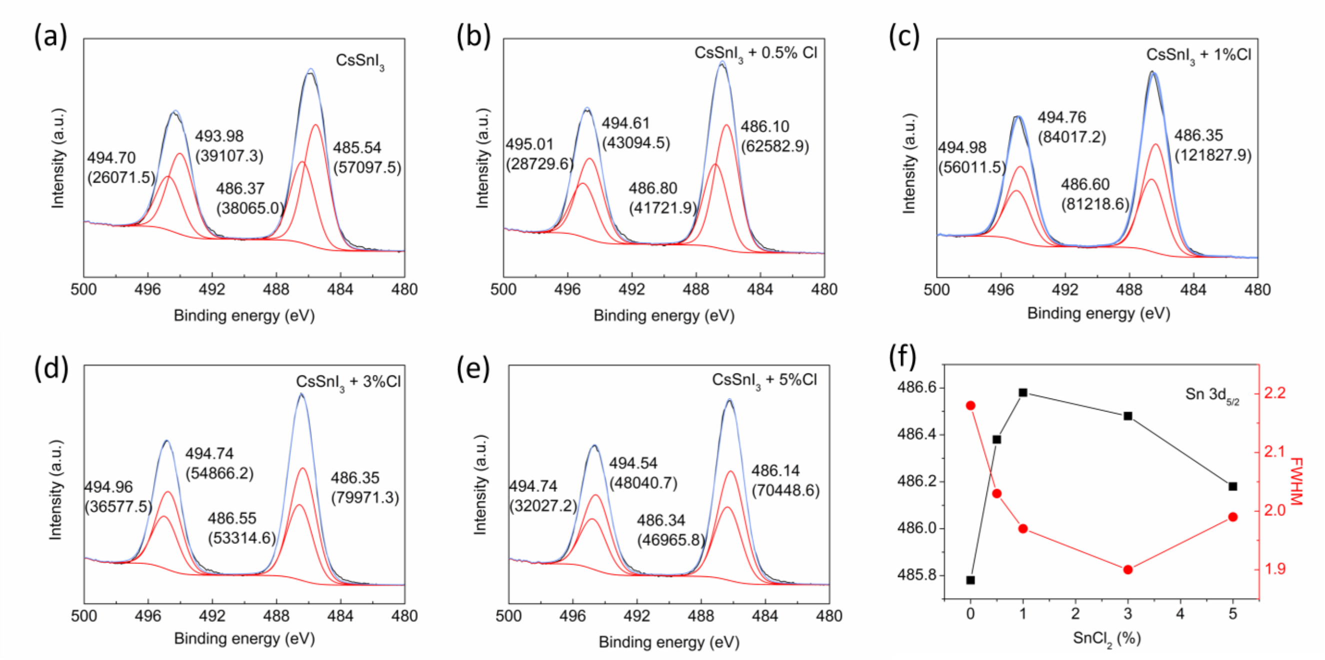

Supplementary Figure 9. Sn 3*d* spectra of $CsSnI_3$ and mixed halide $CsSnI_{3-x}Cl_x$ thin films. (a) to (e) XPS Sn 3*d* spectra of films of $CsSnI_3$ and mixed halide $CsSnI_{3-x}Cl_x$. (f) Sn 3$d_{5/2}$ peak position and FWHM as a function of $SnCl_2$ inclusion.

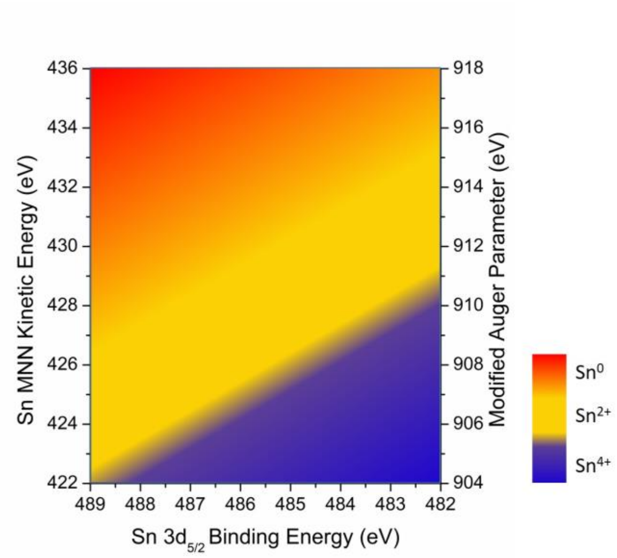

Supplementary Figure 10. Sn 3$d_{5/2}$ and Sn $M_5N_{4,5}N_{4,5}$ Wagner plot. Colour filled with the values from Reference [9], [10], [11].

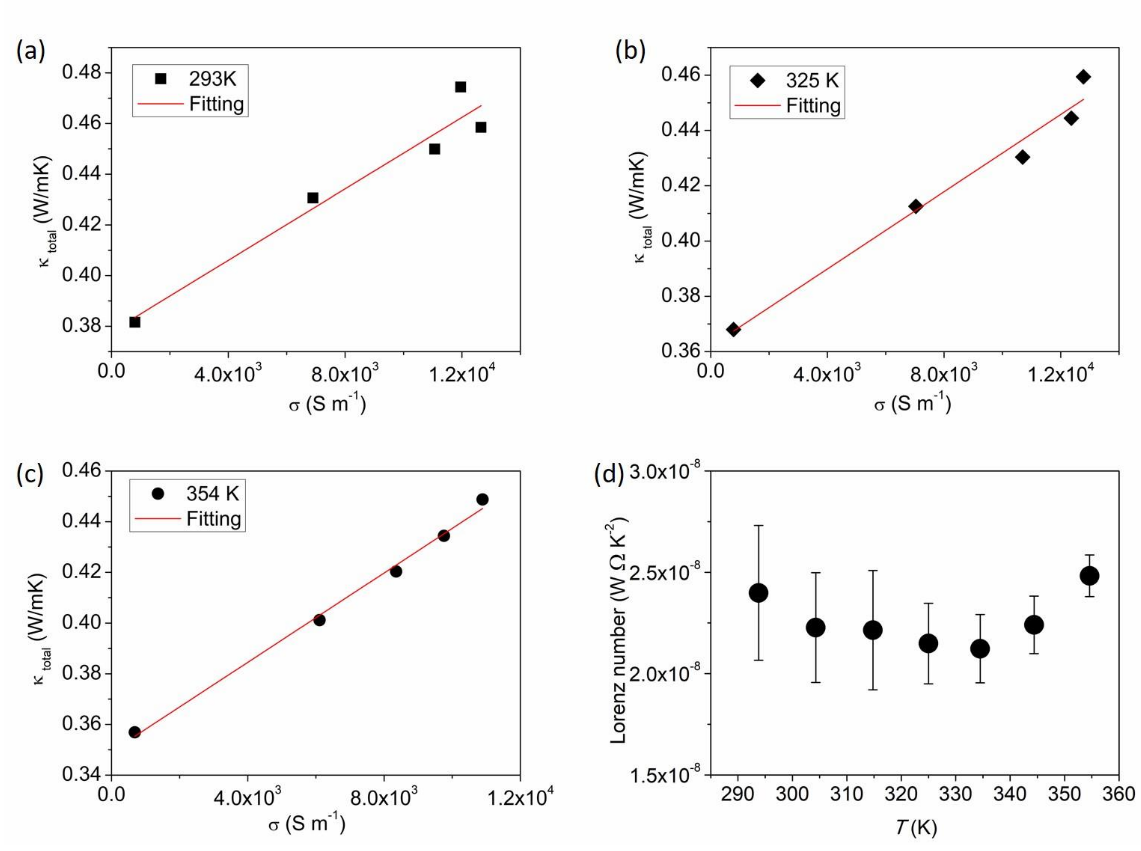

Supplementary Figure 11. Thermal conductivity (total) as a function of electrical conductivity with linear fitting curve at 293 K (a), 325 K (b) and 354 K (c). (d) Temperature dependence of Lorentz number at range of 290 K to 360 K.

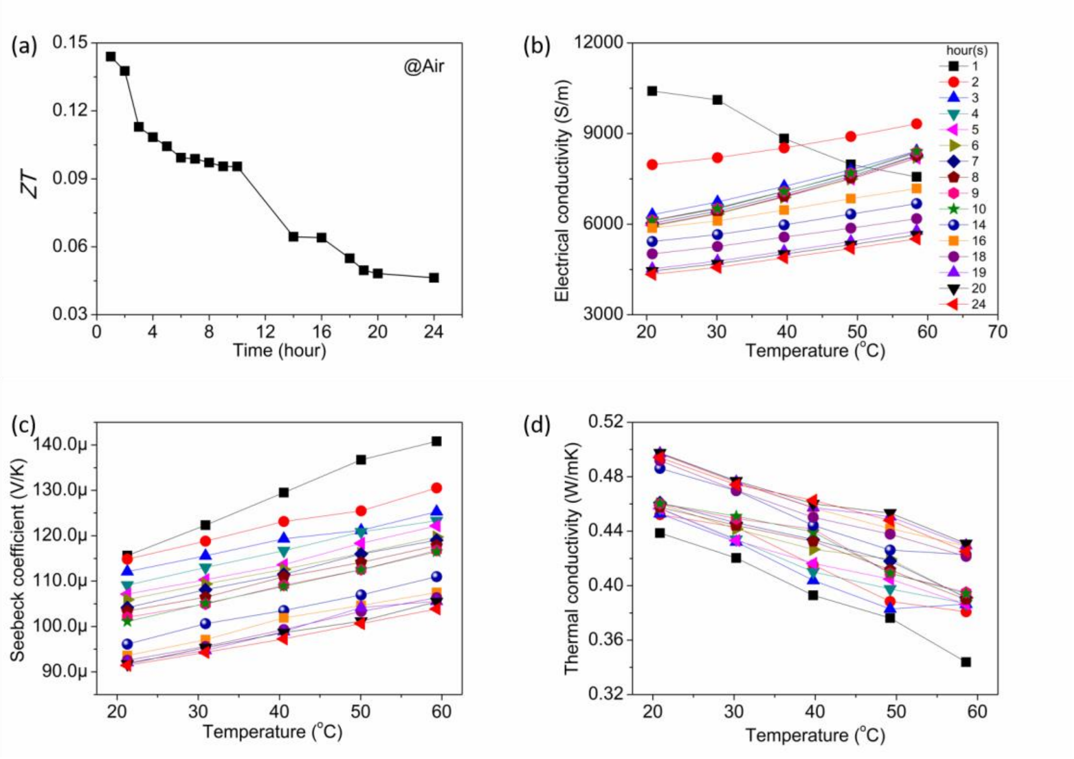

Supplementary Figure 12. Thermoelectric properties stability measurement of 1% doping $CsSnI_{3-x}Cl_x$ perovskite films in air with 40% humidity. (a) figure-of-merit ZT, (b) electrical conductivity, (c) Seebeck coefficient and (d) thermal conductivity.

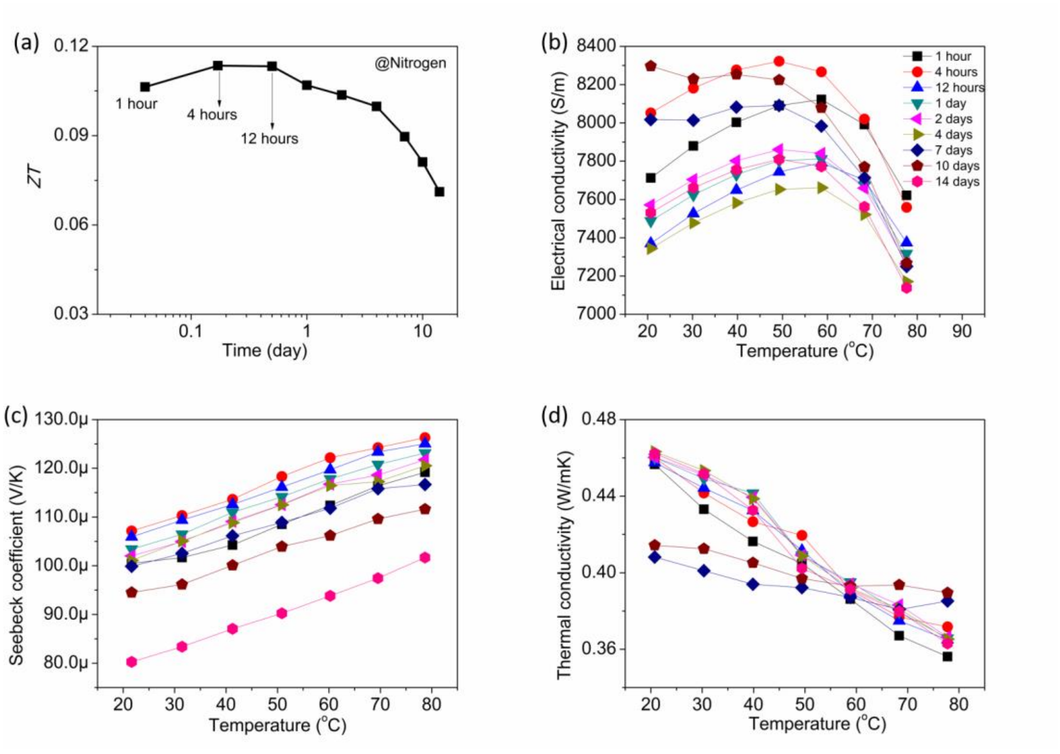

Supplementary Figure 13. Thermoelectric properties stability measurement of 1% doping $CsSnI_{3-x}Cl_x$ perovskite films in nitrogen. (a) Figure of merit , (b) electrical conductivity, (c) Seebeck coefficient and (d) thermal conductivity. It should be noted that samples stored in nitrogen are exposed to air for up to 2 minutes between each measurement due to the transfer time from our instrument to the glovebox and therefor the righthand panel encompasses 18 minutes of air exposure and 10 days of nitrogen storage.

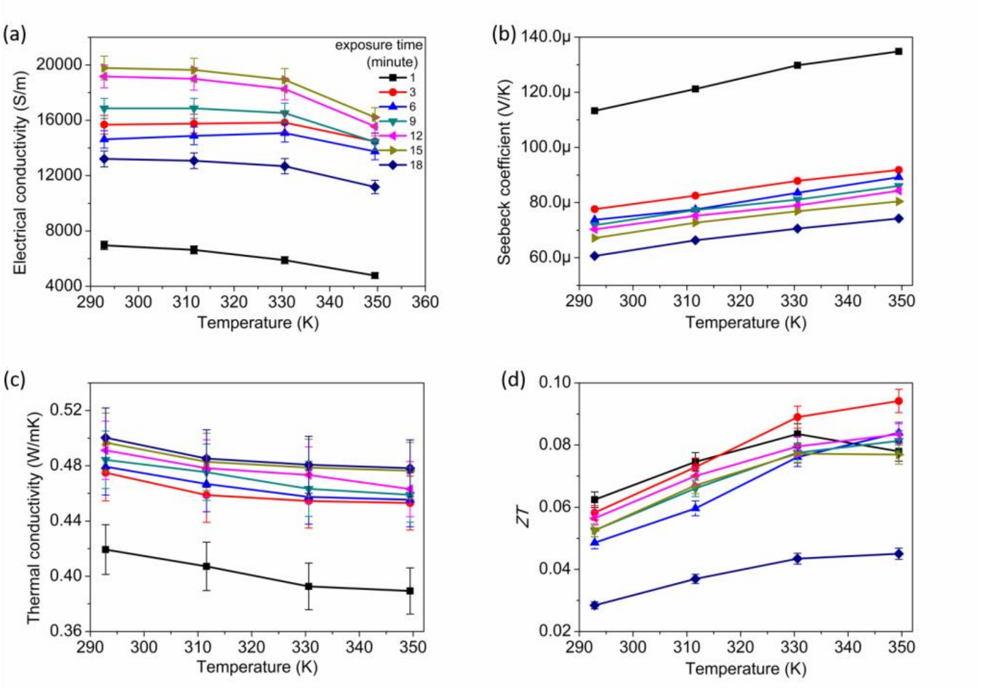

Supplementary Figure 14. Thermoelectric properties of 1% Cl doping $CsSnI_{3-x}Cl_x$ films with thickness of 115 ± 5nm. (a) Electrical conductivity, (b) Seebeck coefficient, (c) thermal conductivity and (d) figure-of-merit ZT.

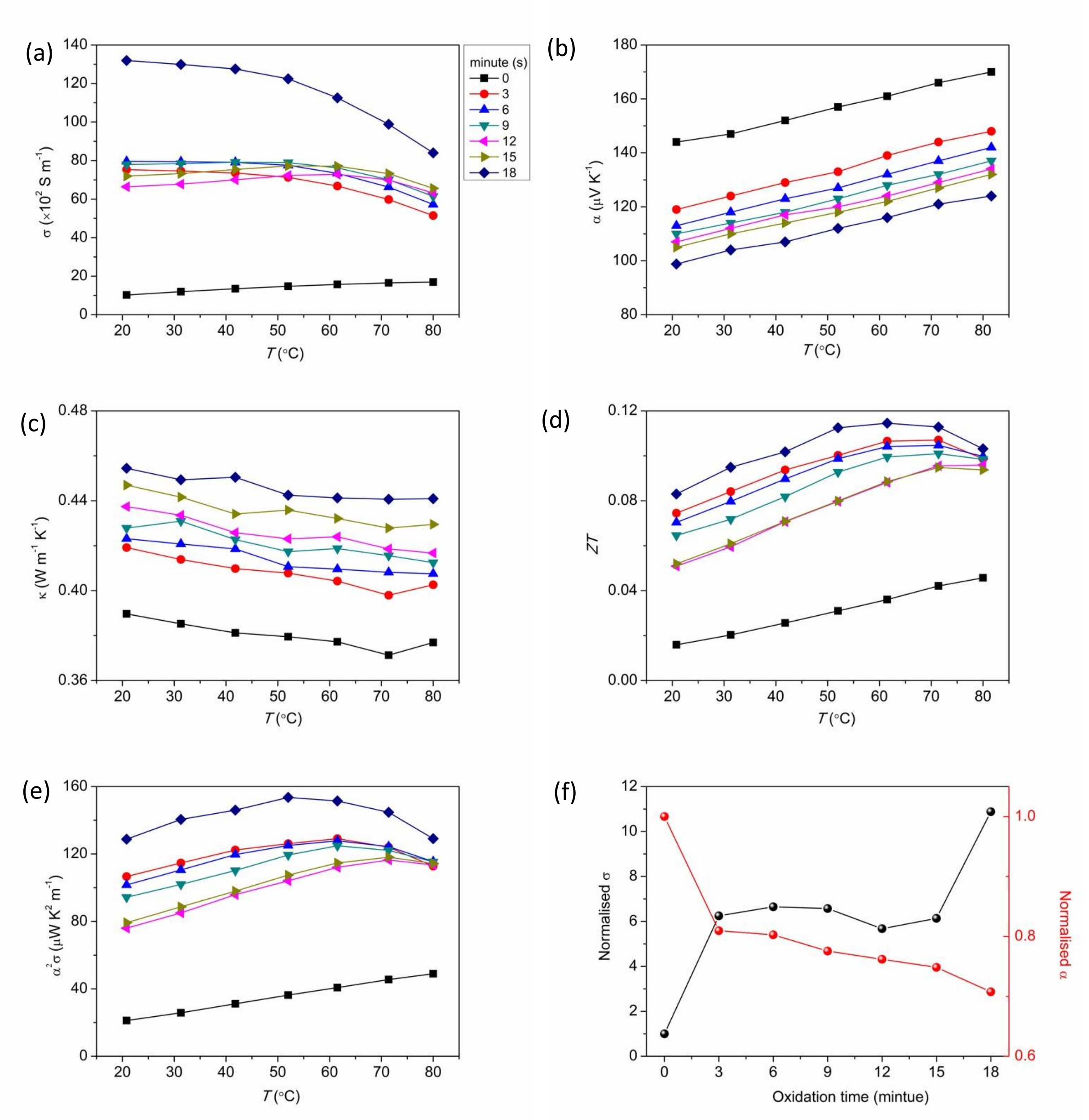

Supplementary Figure 15. Thermoelectric properties of 0.5% $SnCl_2$ mixed $CsSnI_{3-x}Cl_x$ perovskite thin films. The legend in panels (a) refers to the exposure time to air before the measurement and applies to panels (a)-(e).

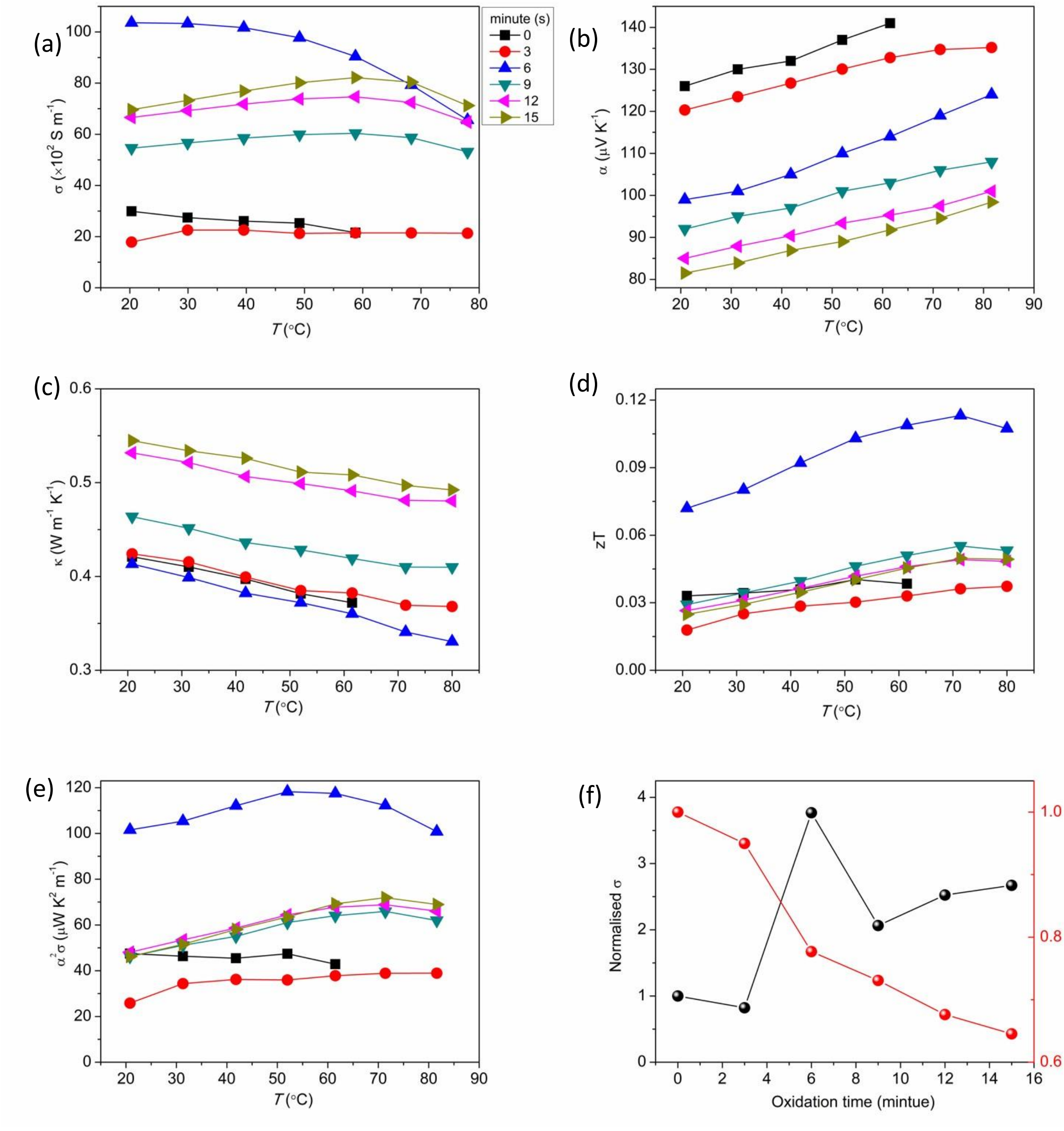

Supplementary Figure 16. Thermoelectric properties of 3% $SnCl_2$ mixed $CsSnI_{3-x}Cl_x$ perovskite thin films. The legend in panels (a) refers to the exposure time to air before the measurement and applies to panels (a)-(e).

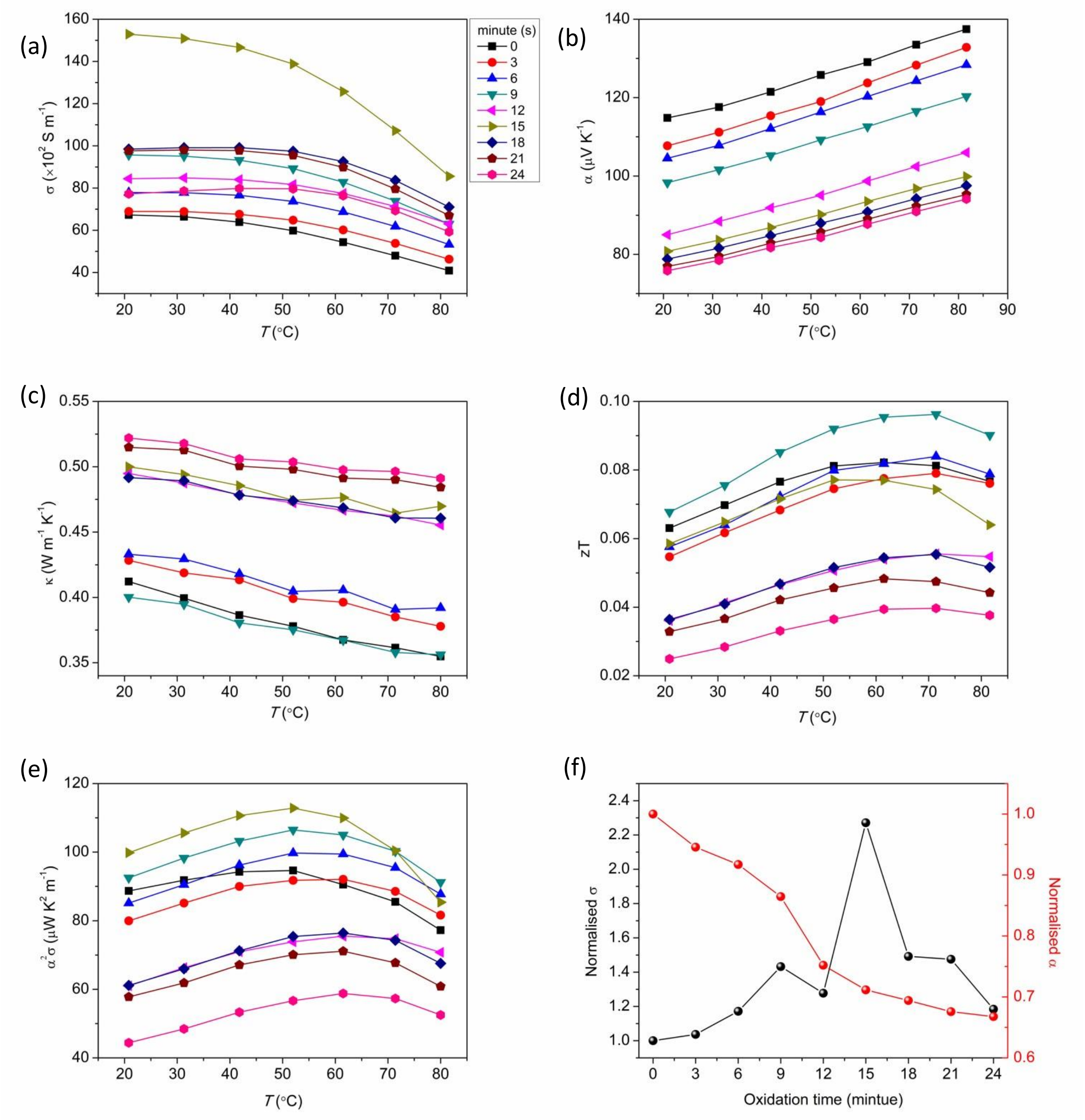

Supplementary Figure 17. Thermoelectric properties of 5% $SnCl_2$ mixed $CsSnI_{3-x}Cl_x$ perovskite thin films. The legend in panels (a) refers to the exposure time to air before the measurement and applies to panels (a)-(e).

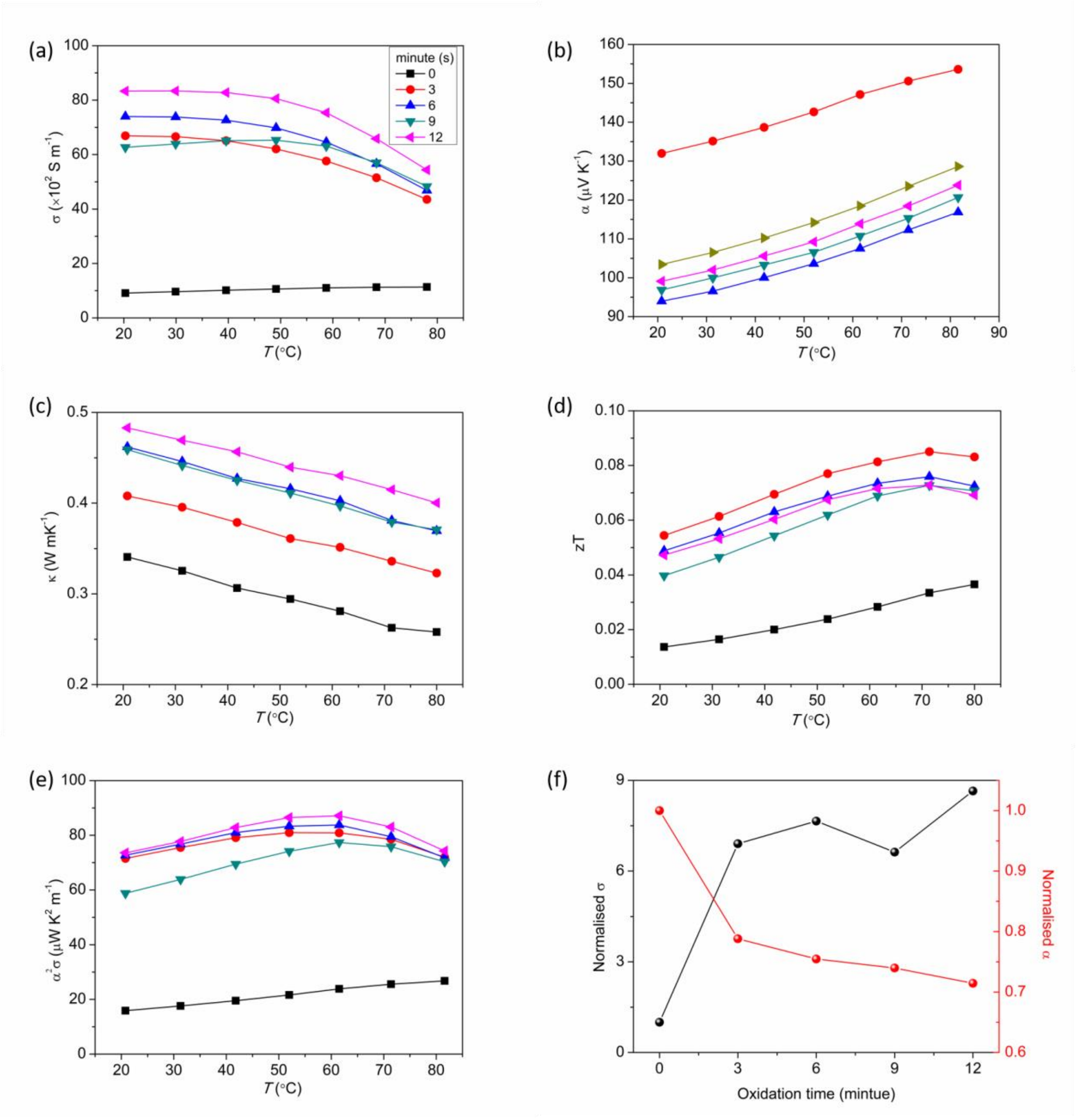

Supplementary Figure 18. Thermoelectric properties of 0% $SnCl_2$ mixed $CsSnI_{3-x}Cl_x$ perovskite thin films. The legend in panels (a) refers to the exposure time to air before the measurement and applies to panels (a)-(e).

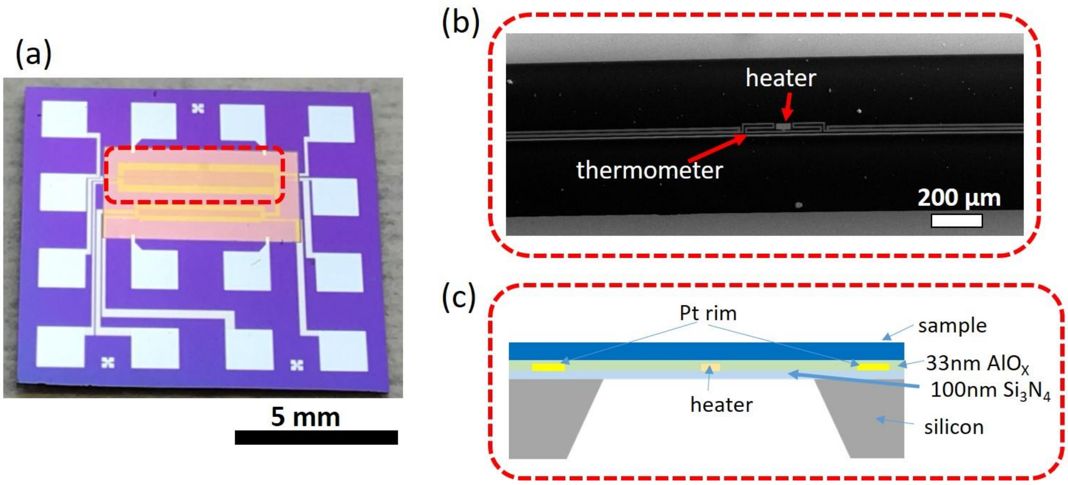

Supplementary Figure 19. (a) Optical image of measurement chip (Linseis Messgeräte GmbH). The pink rectangular area is where the material under test has been deposited. Within the red dashed square area is a suspended film of 100 nm Si_3N_4. (b) SEM image of top view of the suspend membrane with heater and thermometer. (c) Cross-sectional view of the 100 nm Si_3N_4 membrane, framed by a Pt rim to act as a heat sink on the cold side. The heating stripe is positioned along the centre of the membrane for the heating and monitoring temperature. The heating stripe is electrically isolated from the film under test by a 33 nm Al_2O_3 passivation layer.

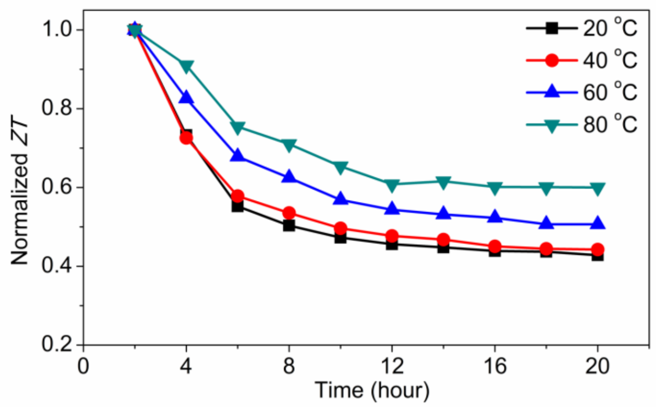

Supplementary Figure 20. Temperature stability of ZT performance measurements in $CsSnI_{3-x}Cl_x$ perovskite films in vacuum.

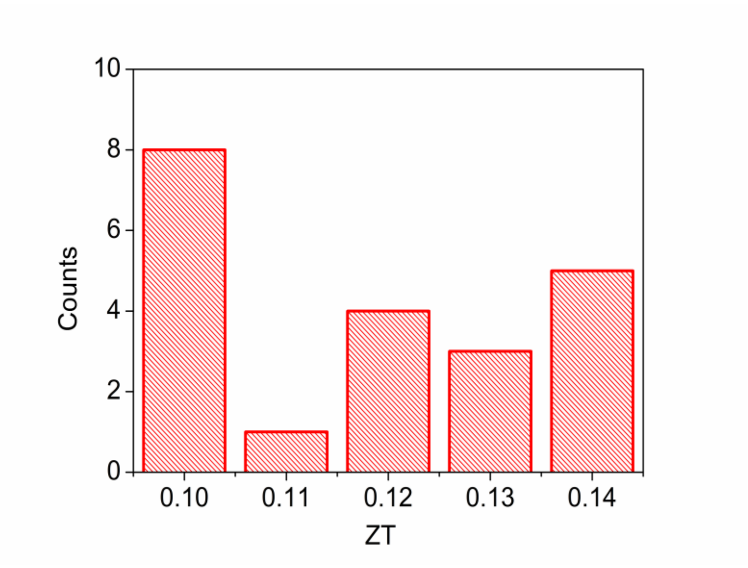

Supplementary Figure 21. The histogram of figure of merit (ZT) in 1% Cl doping $CsSnI_{3-x}Cl_x$ perovskite films.

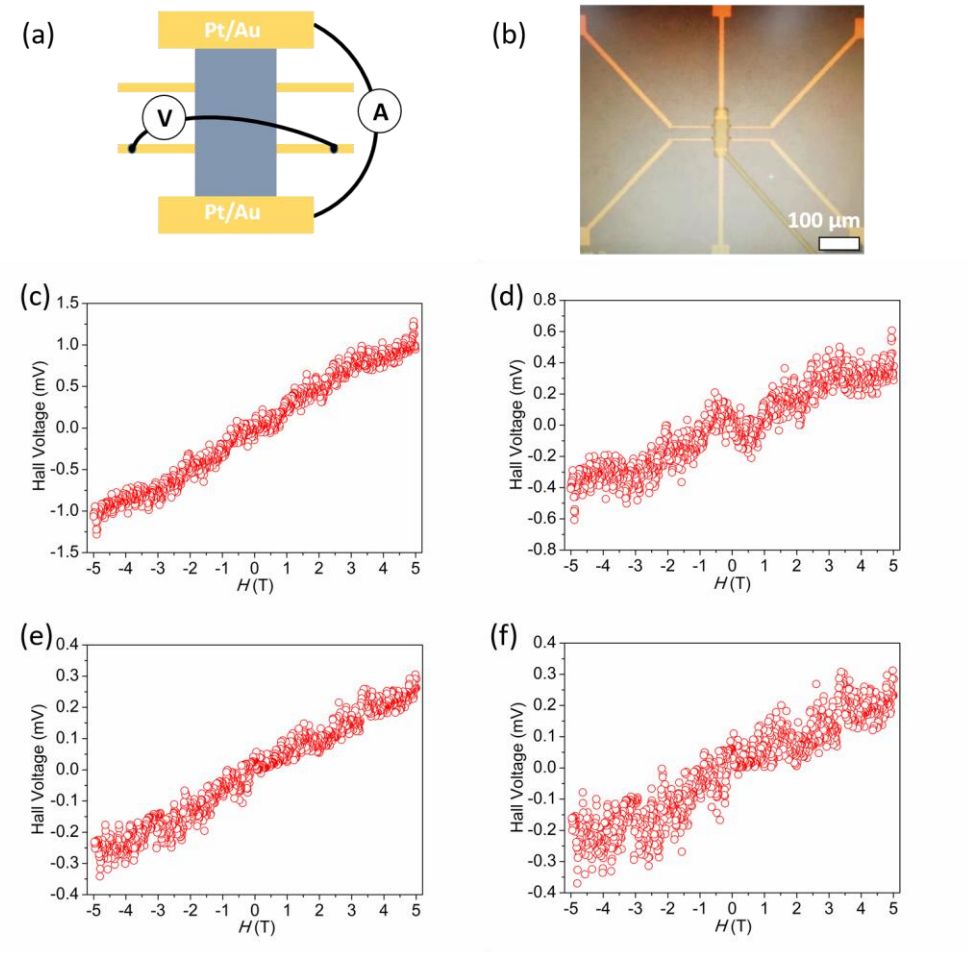

Supplementary Figure 22. Schematic (a) and optical microscopy of the Hall bar architecture (b). Hall voltage *vs* magnetic field *H* in 1% doping $CsSnI_{3-x}Cl_x$ perovskite films for different air exposure time (c) 1 minutes, (d) 6 minutes, (e) 9 minutes and (f) 12 minutes, respectively.

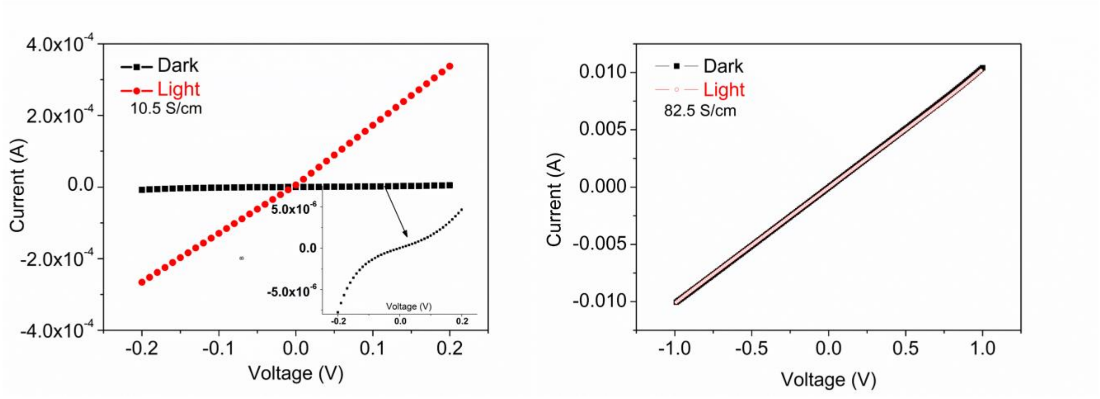

Supplementary Figure 23. I-V measurement of photodetector in the dark and under AM1.5G illumination. Left: encapsulated sample without prior air exposure. Right: sample kept in ambient air for 20 minutes before measurement.

Supplementary Table 1. Sn metal (Sn^0) Auger peaks.

(eV)	$M_5N_{4,5}N_{4,5}$					$M_4N_{4,5}N_{4,5}$	
	$^1S_0$	$^1G_4$	$^3P_2$	$^3F_{2,3}$	$^3F_4$	$^1G_4{}^1D_2$	$^3F_{2,3}$
Barlow* Ref. 10	421.3	425.6	426.8	427.9	428.7	434.1	436.3
Pessa Ref. 11	422.9	425.6		427.6		434.1	

* Reference of Sn metal used in this work

Supplementary Table 2. Experimental Sn core binding energies, Auger kinetic energies and Auger parameters in references.

	E_b 3$d_{5/2}$	ΔE_b 3$d_{5/2}$ (oxide-metal)	E_k $M_5N_{45}N_{45}$	ΔE_k (oxide-metal)	α’	Ref.
Sn^0	483.8	0	431.6	0	915.4	10
Sn^0	484.9	0	430	-	914.9	9
Sn^0	484.65	-	-	-	-	8
Sn^{2+}	486.4	1.5	426.2	3.8	912.6	9
Sn^{2+}/Sn^{4+}	485.7	1.05	425	-	910.7	8
Sn^{4+}	487.1	2.2	424.2	5.8	911.3	9
Sn^{4+}	486.4	1.75	423.3	-	909.7	8

Supplementary Table 3. Sn core binding energies, Auger kinetic energies and Auger parameters in references in this work.

Depth (nm)	E_b $3d_{5/2}$	*ΔE_b $3d_{5/2}$ (oxide-metal)	E_k $M_5N_{45}N_{45}$	*ΔE_k (oxide-metal)	α’
0	486.77	1.87	425.35	4.65	912.12
2.5	486.11	1.21	426.84	3.16	912.95
5	486.2	1.3	426.86	3.14	913.6
7.5	486.06	1.16	427.72	2.28	913.78
10	485.97	1.07	428.6	1.4	914.57

*the energy shift of binding energy and Auger kinetic energy as compared to Sn metal state in Ref 9.

Supplementary Table 4. A summary of thermoelectric performance in halide hybrid perovskite materials.

	Temperature (K)	materials/ methods	dopant	Figure-of-merit, ZT	reference
$CH_3NH_3SnI_3$	295	bulkcrystals	N/A	~ 10^{-3}	1
$CH_3NH_3PbI_3$	295	bulkcrystals	Light	~ 10^{-7}	
$CsSnI_3$	320	nanowires	N/A	0.11	2
$CsSnI_3$	295	thin films	N/A	0.137	3
$CH_3NH_3SnI_3$	300	simulation	n-type<br>p-type	1.7<br>1.1	4
$CH_3NH_3PbI_3$	300	simulation	n-type<br>p-type	1.2<br>0.8	
$C_6H_4NH_2CuBr_2I$	363	simulated κ<br>thin films	N/A	0.22	5
$CsSnI_3$	345	thin films	$SnCl_2$	0.14	Our work

Supplementary Note 1

Optical images of $CsSnI_3$ thin films.

Optical images Supplementary Figure 1 (A) to (E), show that all the films formed by vacuum deposition present a "mirror-like" surface. Films formed from the co-evaporation method directly show a perovskite black phase, whereas the sequentially deposited and SLS films are red-brown (due to the coexistence of SnI_2 and CsI layers in the film) but convert to a black phase upon baking. The B-γ-$CsSnI_3$ undergoes a significant phase transition to the Y-phase (yellow phase) when exposed to air. The Y-phase will continuously change to a dark-green phase (Cs_2SnI_6) where Sn^{2+} is totally oxidised to Sn^{4+}. The Cs_2SnI_6 films are semi-transparent and stable in air.

Supplementary Note 2

XRD structure characterization of seed layer plus sequential deposition (SLS) mixed halide $CsSnI_{3-x}Cl_x$ thin films.

We performed X-ray diffraction analysis of SLS 5% $SnCl_2$ mixed halide $CsSnI_{3-x}Cl_x$ thin films, which showed (Supplementary Figure 2 (a)) peaks at 25.02° and 29.15°, corresponding to (220) and (202) planes respectively of the orthorhombic B-γ-$CsSnI_3$ crystal structure. In fact, the mixed halide films processed by SLS show a similar crystal structure to undoped SLS $CsSnI_3$ films (XRD presented in the main text). To investigate the difference between the pure $CsSnI_3$ and mixed $CsSnI_{3-x}Cl_x$, we performed a slow X-ray diffraction scan from 20° to 30° at a rate of 1°/minute (Supplementary Figure 2 (b)). A peak at 23.00° is clearly observed, which is different to the typical peak at 22.80° of the $CsSnCl_3$ perovskite (011) plane and is also shifted slightly with respect to the weak $CsSnI_3$ perovskite (213) plane at 22.94°.[6] Supplementary Figure 3 (a-b) shows that the main change to the spectrum after exposure to air for 30 minutes is the (103) at 32.9°. The other peaks are reasonably unaffected.

Supplementary Note 3

Quantitative analysis of Cl states in mixed halide perovskite by X-ray photoelectron spectroscopy (XPS).

To investigate the Cl bonding environment in the films, we performed X-ray photoelectron spectroscopy (XPS) of Cl 2*p* in 0.5%, 1%, 3% and 5% $SnCl_2$ mixed halide $CsSnI_{3-x}Cl_x$ perovskite films. The percentage we use refers to the mass of $SnCl_2$ relative to SnI_2 in our thin films before the baking step. The final atomic % of Cl in the film will be much lower. As shown in Supplementary Figure 6, the fitted Cl $2p_{3/2}$ peaks in doped films have an energy shift compared to Marshall's work[7] (Cl $2p_{3/2}$:198.20 eV), where a $SnCl_2$ ad-layer is formed on top of a pure $CsSnI_3$ perovskite structure without incorporation of the chloride ions into the perovskite structure. The line shape of Cl 2*p* spectra is broader compared with the Cl 2*p* spectra of $SnCl_2$,[7] which is probably due to the mix of environments Cl experiences in the mixed halide structure. Moreover, we quantified the Cl doping profile in our mixed halide $CsSnI_{3-x}Cl_x$ (1% $SnCl_2$) films by XPS (Supplementary Figure 6), finding that Cl is present in the top layer only, and diffusing just a few nanometres into the bulk.

Supplementary Note 4

UV-vis absorption spectra for air stability analysis.

We quantified the film stability in ambient air by following the quenching of the optical absorbance. The sequentially deposited films presented a poor stability where the intensity of a degradation peak at 680 nm gradually increase after 100 minutes air exposure. For the co-evaporated films, there was no clear peak at 680nm after 500 minutes air exposure though the absorbance at 420 nm reduced to 32% of its original value. In the SLS films, the degradation peak at 680 nm presented from ~380 minutes, and, after 500 minutes, the absorbance at 420 nm had reduced to 41% of its original value. When $SnCl_2$ was introduced to form the mixed halide $CsSnI_{3-x}Cl_x$, the large improvement in film stability evident from UV-vis absorption spectra that show no emergence of the degradation peak at 680 nm even after 500 minutes (Supplementary Figure 8 g and h). In Supplementary Figure 8(i), sequentially deposited films (red) show a poor stability with 60% reduction in absorption at 420 nm after 100 minutes air exposure. Mixed halide $CsSnI_{3-x}Cl_x$ perovskite films with 5% $SnCl_2$ show the best stability among all with just 3% quenching of the 420 nm peak after 100 minutes air exposure.

Supplementary Note 5

Sn oxidation states in $CsSnI_{3-x}Cl_x$ perovskite films.

For the pristine $CsSnI_3$ perovskite films, the binding energy of Sn $3d_{5/2}$ was observed at 485.8 eV. However, the binding energy of Sn $3d_{5/2}$ in mixed halide $CsSnI_{3-x}Cl_x$ samples had an energy shift of up to 0.8 eV with respect to the pristine $CsSnI_3$, confirming a change in the Sn local chemical environment in the upper layers of the perovskite films as a result of Cl-incorporation. The full width at half maxima (FWHM) of the Sn $3d_{5/2}$ peaks decrease with Cl-doping (Supplementary Figure 7 (f)), from the pristine $CsSnI_3$ value (~ 2.18 eV) to a minimum at 3% $SnCl_2$ $CsSnI_{3-x}Cl_x$ (~ 1.90 eV).

Supplementary Note 6

Auger electron spectroscopy (AES) of Sn.

The reported Sn 3*d* core binding energy has shifts between Sn^0, Sn^{2+} and Sn^{4+} oxidation states in the range 1 – 1.8 eV (Sn^0 to Sn^{2+}) and 1.8 – 2.5 eV (Sn^0 to Sn^{4+}), respectively.[8,9] The small shift of 0.4 eV from Sn^{2+} to Sn^{4+} makes quantitative analysis of oxidation in our films challenging, AES is employed to distinguish Sn^{2+} and Sn^{4+} states due to substantial spectral shifts. Supplementary Table 1 shows the AES peaks of Sn metal.[10,11] Upon oxidation of metallic Sn samples, Kövér found Sn MNN kinetic energy shift of ~3.8 eV, 5.8 eV for SnO and SnO_2.[9] Lee observed small shifts ~2 eV for 0.4 monolayer (ML) and 3.4 ML Sn oxides attributed to the formation of SnO rather than SnO_2.[12]

Another indicator of Sn chemical state, the modified Auger parameter, is shown in Supplementary Table 2[3,4,5] and 3 (this work). The coloured Wagner plot (Supplementary Figure 10), shows the boundary coordinates of the oxidation states and where there is a coexistence between two states (i.e. between Sn^0 (red) and Sn^{2+} (yellow) or Sn^0 and Sn^{2+}). In Supplementary Table 3, α' is 912.12 eV, 912.95 eV, 913.6 eV,913.78eV and 914.57 eV for 0, 2.5, 5, 7.5 and 10 nm depth, respectively. Comparing with α' of Sn^0 (915 eV)[9,12] and Sn^{4+} (911.2 eV)[8] it is clear that our films contain oxidised forms of Sn in a mix of Sn^{2+} and Sn^{4+} states. As the depth goes down to 10 nm, there is a reduction in the Sn^{4+} character of the perovskite until something resembling pure Sn^{2+} is reached. This combined with the spectral fitting in the main paper (Figure 4a,b) we can conclude that on the timescale of our thermoelectric measurements, the oxidation process only proceeds in the outer surface layer of our films, leaving the bulk in a pristine state.

Supplementary Note 7

Wiedemann-Franz law in $CsSnI_{3-x}Cl_x$ perovskite semiconductor thin films and extraction of the Lorenz number.

The Wiedemann-Franz law (WFL), $\kappa_e = \sigma TL$, connects the electronic component of the thermal conductivity κ_e to the electrical conductivity, σ, of a material, where L is Lorenz number and T is the temperature. In the free electron model, L is given by a constant, $L = \frac{\pi^2}{3}\left(\frac{k_B}{e}\right)^2 = 2.44 \times 10^{-8}\mathrm{W\Omega K^{-2}}$. In most systems L takes a value close to this one, however, deviation of L from this value has been reported in many systems, such as organic crystals,[13] heavy fermion compounds[14] and disordered Luttinger liquids[15]. Extraction of an accurate value of L is important especially in thermoelectric systems where it impacts the optimisation of ZT.

Thermal conductivity can therefore be deviated into two parts: lattice and electron contribution, as following:

$\kappa = \kappa_{lattice} + \sigma LT$ (1)

We can extract L and lattice thermal conductivity, $\kappa_{lattice}$, by plotting thermal conductivity versus electrical conductivity for a number of experimental measurements. To do this, after the first measurement of the samples, it was exposed to air for 3 minutes before re-measuring the electrical and thermal conductivity (σ_{3mins} and κ_{3mins}). Then the same sample was exposed to air for an additional 3 minutes exposure, before measuring the electrical and thermal conductivity again (σ_{6mins} and κ_{6mins}). Thus, we obtained several values (σ_{time} and κ_{time}) and plot σ_{time} vs. κ_{time}. From the equation, $\kappa_{time} = \kappa_{lattice} + \sigma_{time}LT$, we see that the slope gives Lorentz number times temperature (L*T) and the intercept is lattice thermal conductivity.

In this situation, we must approximate that the lattice thermal conductivity does not change as the dopants are introduced. In fact, dopants are defects, and do influence the lattice structure and disorder. Consequently, quantification of the Lorenz number is challenging in many thermoelectric materials. In our case, $CsSnI_3$ perovskites support an effective way to extract L because of the self-doping process, which does not introduce extrinsic dopants. The Sn^{4+} sites that act as self-dopants are

shown in the main manuscript to be located at the top surface of the film only (in a layer < 10 nm thick), but provide free charges to the bulk (~300 nm thick). The lattice thermal conductivity in > 95 % of the film thickness is therefore unaffected by the doping process. As shown in Supplementary Figure 11 (a), (b), and (c), the fitting curve gives two parameters: slope (LT) and intercept ($\kappa_{lattice}$) and errors are given by the error on the fit.

Supplementary Note 8

Thermoelectric property measurement details.

Electrical conductivity measurement is based on the van der Pauw method with four needle-like contact pads at the four corners of the material under test, as shown in Supplementary Figure 16 (a). Electrical conductivity can be calculated by the following equation:

$$\exp(-\sigma\pi d \times R_{vertical}) + \exp(-\sigma\pi d \times R_{horizontal}) = 1 \qquad (2)$$

where d is film thickness, R is resistance of the films with vertical and horizon directions (as pictured). The thin-film heater and thermometer are located along the centre-line of the membrane, and the temperature gradient can be adjusted with the current in heater. The cold side temperature is taken as the chip temperature (measured underneath in the base holder), allowing the thermovoltage to be obtained and a Seebeck coefficient calculated. In-plane thermal conductivity is be measured by a transient 3-ω method. An alternating current $I(t) = I_0 \times \cos(\omega t)$ is used to heat the stripe. Joule heating occurs at frequency 2ω due to the heating power, $I_\omega^2 = I_0^2 R(1 + \cos(2\omega t))/2$. As a result, the temperature oscillates at 2ω, and the temperature dependent electrical resistance also has a component at 2ω ($R_{2\omega}$). The temperature change of the heating stripe is measured with a lock-in amplifier which can extract the third harmonic of the voltage drop across the heating stripe ($V_{3\omega} = I_\omega R_{2\omega}$). The third-harmonic voltage captures the second-harmonic temperature rise ($\Delta T_{2\omega}$) in the heater, which is a function of the thermal properties of the underlying materials. Considering in-plane thermal conductivity, by solving the two dimensional partial differential heat equation across the membrane with the given boundary conditions, the general expression for the amplitude of the 3ω oscillation $V_{3\omega}$ is expressed as[16]:

$$|V_{3\omega}| = \frac{\beta R^2 I_0^3}{4(\frac{2\kappa t l}{w})\sqrt{1+\omega^2(4\tau^2+\frac{w^4}{24D^2}+\frac{\tau w^2}{3D})}} \qquad (3)$$

where β is the temperature coefficient of resistance, t is the total thickness of sample plus 100nm of Si_3N_4 membrane and 33 nm of Al_2O_3, l is the length of the heater, w is the width of the membrane, τ is the thermal relaxation time, $D = \kappa/\rho c$, where ρ is the mass density and c is the specific heat capacity.

When using low frequencies, the measurement can be performed at quasi-steady state conditions, where the ω becomes negligible and $V_{3\omega}$ becomes constant. Equation 3 can be written as:

$$V_{3\omega} = \frac{\beta R^2 I_0^3}{4\left(\frac{2\kappa t l}{w}\right)} \qquad (4)$$

To get the thermal conductivity of the sample, the contribution of membrane thermal conductance should be removed. The sample thermal conductivity is given by

$$\kappa_{sample} = \frac{\kappa t - \kappa_m t_m}{t_s} \qquad (5)$$

Where κ_m is the membrane thermal conductivity, t_m is the membrane thickness, t_s is the sample thickness. The errors of electrical and thermal conductivity are dominated by the measurement of film thickness. The errors on the Seebeck coefficient come from the fitting error of thermal volatge *vs.* temperature gradient data. Because the film thicknesses used in electrical and thermal conductivity are identical, they cancel out in the calculation of ZT, limiting the error on the final value.

Supplementary Note 9

Thermoelectric properties of 1% Cl doping $CsSnI_{3-x}Cl_x$ thinner films with thickness of 115 ± 5 nm.

We performed thermoelectric property measurements of thinner mixed halide perovskite with thickness of 115 ± 5 nm. The electrical conductivity at room temperature is 168.6 ± 7.2 S cm^{-1} for the sample under air exposure in 9 minutes, which is higher than the highest value in the film with thickness 265 ± 15 nm (126.5 S cm^{-1}, air exposure 9 minutes). At longer air exposure times, the conductivity increases to ~200 S cm^{-1} before degrading, which is significantly higher than any value observed for the thicker films. However, the initial electrical conductivity in thinner films measured in glovebox with no air exposure is 1.8 S cm^{-1}, which is lower compared to the value in thick films (8.6 S cm^{-1}). This trend can be explained as following. Normally, when the film thickness decreased, nanostructuring plays a key role in thermoelectric properties, such as grain boundaries, edge effect and point defects. In this case, the thinner films with increased grain boundaries and surface scattering will result the lower electron mobility than that in thick films, which is consistent with our measurements of initial electrical conductivity. When the films are exposed to air, the oxidation process of Sn^{2+} to Sn^{4+} will initiate in the surface layer. That oxidation process will also happen from the grain boundaries, which are more numerous in the thinner films. With the increasing charge concentration, the transport energy level will shift close to Fermi level, resulting in a reduced Seebeck coefficient.[17,18] Consequently, the Seebeck coefficient of the thin films is in the range 60.6 to 113.3 μV K^{-1}, which is smaller than in thick films (103.0 to 144.7 μV K^{-1}). This is shown in In Supplementary Figure 21 (b), above. As a consequence of the higher electrical conductivity in the thin films, the thermal conductivity (Supplementary Figure 21 (c)) is also higher than in thick films, attributed to the larger electron contribution to thermal transport.

Overall, the highest figure of merit in thinner films is 0.09 ± 0.004 at 350 K with air exposure 3 minutes which is lower than the maximum of 0.14 measured for the thicker films. We can therefore say that although the different morphology of the thin films is conducive to better electrical conductivity, this does not translate into improved thermoelectric figure of merit, ZT.

Supplementary Note 10

Details of Hall effect measurements in $CsSnI_{3-x}Cl_x$ perovskite thin films.

We performed Hall effect measurements on $CsSnI_{3-x}Cl_x$ perovskite thin films in a Hall bar architecture. The perovskite films were deposited by SLS method on the pre-patterned substrates with electrodes of 15 nm Pt and 75 nm Au. The perovskite films were then light graphically patterned to a precised Hall bar geometry following the procedure reported elsewhere.[19] The magnetic field was applied perpendicular to the sample plane with a sweep rate of 0.2 T/min from 5 T to -5 T. All the measurements were performed at room temperature.

Supplementary Note 11

Photoconductivity in $CsSnI_{3-x}Cl_x$ thin films.

We performed electrical conductivity measurements of $CsSnI_{3-x}Cl_x$ perovskite films both in dark and under AM1.5G illumination. To do this, a photodetector was fabricated by depositing $CsSnI_{3-x}Cl_x$ films on silicon wafer (SiO_2/Si). 80 nm Au was deposited through a shadow mask to form top contacts. Current voltage curves were measured using a Keithley 2400 sourcemeter, and AM 1.5G solar illumination at 100 mW cm^{-2} (1 sun) was used as light source. As shown in Supplementary Figure 23, there was a light response in encapsulated samples without air exposure. Electrical conductivity changed from 0.8 S/cm in dark to 10.5 S/cm upon simulated solar illumination. However, for samples with 20 minutes air exposure, the current was identical in the dark and under illumination at a higher value of 82.5 S/cm. Charge carriers introduced by solar irradiation were therefore deemed to be of negligible effect in high conductivity $CsSnI_{3-x}Cl_x$ semiconductors.

Supplementary References

1 Mettan, X. *et al.* Tuning of the Thermoelectric Figure of Merit of $CH_3NH_3MI_3$ (M=Pb,Sn) Photovoltaic Perovskites. *J. Phys. Chem. C* **119**, 11506-11510, (2015).

2 Lee, W. *et al.* Ultralow Thermal Conductivity in All-Inorganic Halide Perovskites. *P. Natl. Acad. Sci. USA* **114**, 8693-8697 (2017).

3 Saini, S., Baranwal, A., Yabuki, T., Hayase, S., & Miyazaki, K. Growth of Halide Perovskites Thin Films for Thermoelectric Applications. *MRS Advances*, 4(30), 1719-1725, (2019)

4 He, Y. P. & Galli, G. Perovskites for Solar Thermoelectric Applications: A First Principle Study of $CH_3NH_3Al_3$ (A = Pb and Sn). *Chem. Mater.* **26**, 5394-5400, (2014).

5 Liu, Y. C., Li, X. L., Wang, J. B., Xu, L. & Hu, B. An Extremely High Power Factor in Seebeck Effects Based on A New N-Type Copper-Based Organic/Inorganic Hybrid $C_6H_4NH_2CuBr_2I$ Film with Metal-Like Conductivity. *J. Mater. Chem. A* **5**, 13834-13841 (2017).

6 Chung, I. *et al.* $CsSnI_3$: Semiconductor or Metal? High Electrical Conductivity and Strong Near-Infrared Photoluminescence from a Single Material. High Hole Mobility and Phase-Transitions. *J. Am. Chem. Soc.* **134**, 8579-8587 (2012).

7 Marshall, K. P., Walker, M., Walton, R. I. & Hatton, R. A. Enhanced Stability and Efficiency in Hole-Transport-Layer-Free $CsSnI_3$ Perovskite Photovoltaics. *Nat. Energy* **1**, 2016.178 (2016).

8 Asbury, D. A. & Hoflund, G. B. A Surface Study of the Oxidation of Polycrystalline Tin. *J. Vac. Sci. Technol. A* **5**, 1132-1135 (1987).

9 Kover, L. *et al.* Electronic-Structure of Tin Oxides-High-Resolution Study of Xps and Auger-Spectra. *Surf. Interface Anal.* **23**, 461-466 (1995).

10 Barlow, S. M., Bayatmokhtari, P. & Gallon, T. E. $M_{4,5}N_{4,5}N_{4,5}$ Auger Spectrum of Tin and Oxidized Tin. *J. Phys. C Solid State* **12**, 5577-5584 (1979).

11 Pessa, M., Aksela, S. & Karras, M. New Fine Structure in Elctron-Excited Auger Spectra from Solid Surfaces. *Phys. Lett. A* **31**, (1970).

12 Lee, A. F. & Lambert, R. M. Oxidation of Sn Overlayers and the Structure and Stability of Sn Oxide Films on Pd(111). *Phys. Rev. B* **58**, 4156-4165 (1998).

13 Casian, A. Violation of the Wiedemann-Franz Law in Quasi-One-Dimensional Organic Crystals. *Phys. Rev. B* **81,** (2010).

14 Tanatar, M. A., Paglione, J., Petrovic, C. & Taillefer, L. Anisotropic Violation of the Wiedemann-Franz Law at A Quantum Critical Point. *Science* **316**, 1320-1322 (2007).

15 Garg, A., Rasch, D., Shimshoni, E. & Rosch, A. Large Violation of the Wiedemann-Franz Law in Luttinger Liquids. *Phys. Rev. Lett.* **103,** (2009).

16 Linseis, V., Volklein, F., Reith, H., Woias, P. & Nielsch, K. Platform for In-Plane ZT Measurement and Hall Coefficient Determination of Thin Films in A Temperature Range from 120 K up to 450 K. *J. Mater. Res.* **31**, 3196-3204 (2016).

17 Lu, N. D., Li, L. & Liu, M. A Review of Carrier Thermoelectric-Transport Theory in Organic Semiconductors. *Phys. Chem. Chem. Phys.* **18**, 19503-19525 (2016).

18 Germs, W. C., Guo, K., Janssen, R. A. J. & Kemerink, M. Unusual Thermoelectric Behavior Indicating a Hopping to Bandlike Transport Transition in Pentacene. *Phys. Rev. Lett.* **109** (2012).

19 Chang, J. F. *et al.* Hall-Effect Measurements Probing the Degree of Charge-Carrier Delocalization in Solution-Processed Crystalline Molecular Semiconductors. *Phys. Rev. Lett.* **107** (2011).
